# Pneumomediastinum as a complication of emphysematous cholecystitis: Case report

**DOI:** 10.1186/1471-230X-10-99

**Published:** 2010-09-02

**Authors:** Luciano Delgado-Plasencia, Ignacio González-García, Diana Rodríguez-González, Esther Torres-Monzón A

**Affiliations:** 1 Department of General Surgery, University Hospital of Canary Islands. Ofra s/n, La Cuesta, Santa Cruz de Tenerife, 38320, Spain; 2 Department of Radiology, University Hospital of Canary Islands, Ofra s/n, Santa Cruz de Tenerife, 38320, Spain

## Abstract

**Background:**

Emphysematous cholecystitis is a variant of acute cholecystitis which is generally caused by gas-forming organisms. Emphysematous cholecystitis may cause gas spreading within the subcutaneous tissue, peritoneal cavity and retroperitoneum.

**Case presentation:**

We present a case of emphysematous cholecystitis in a middle-aged diabetic patient who, postoperatively, presented edema in both flanks and left chest crepitation on palpation, associated with hemodynamic worsening. Computed tomography scan of the chest and abdomen revealed a large pneumomediastinum, pneumoretroperitoneum, gas in subcutaneous tissue and flank abscesses. In both blood and surgical wound exudate cultures, *Escherichia coli *was found.

**Conclusion:**

Emphysematous cholecystitis should be considered as a possible cause of pneumomediastinum.

## Background

Emphysematous cholecystitis (EC) is a rare life-threatening form of acute cholecystitis presenting mainly in patients aged 50-70 years. The rate of male-female incidence is 3-8/1 [1], while the mortality rate due to EC is reported as 15% compared to 4% for acutecholecystitis [1,2].

Approximately 50% of patients have diabetes mellitus and suffer other types of associated pathology such as peripheral vascular disease [2]. In these patients, EC frequently occurs without severe symptoms.

EC is widely considered to be caused by a gas-forming micro-organism in general. The gas may disseminate to subcutaneous tissue, as well as to the peritoneal and retroperitoneal cavity [3]. We present a case of association between EC and pneumomediastinum.

## Case Presentation

A 53 year-old man with type II diabetes mellitus was admitted to our Emergency Department with upper right quadrant abdominal pain. He was feverish, had experienced discomfort during three days, and showed hemodynamic instability. Physical examination revealed muscular defense of the upper abdomen.

Laboratory tests showed elevated white blood cell count (13500/mm3, 95% neutrophyls), prothrombin activity: 53%, C-reactive protein >90 mg/L, erythrocyte sedimentation rate: 97/mm3 and blood sugar of 298 mg/dl. Chest X-ray showed no pulmonary infiltrates. Ultrasonography showed multiple lithiasis and thickening of the gallbladder wall with hyperechogenic points suggestive of intramural gas. Based on these findings, a diagnosis of EC was made.

Emergency open cholecystectomy revealed hydropic gallbladder, length approximately 10 cm, with edematous, gangrenous walls, greater omentum adhered to the surface, microlithiasis, and purulent content. The rest of the abdominal cavity showed no macroscopic signs of purulence. Postoperatively, a picture of sepsis was detected requiring amines and treatment with piperacillin-tazobactam. At five days after intervention, the patient presented edema in both flanks and left thorax crepitation on palpation, associated with hemodynamic worsening.

**Figure 1:**
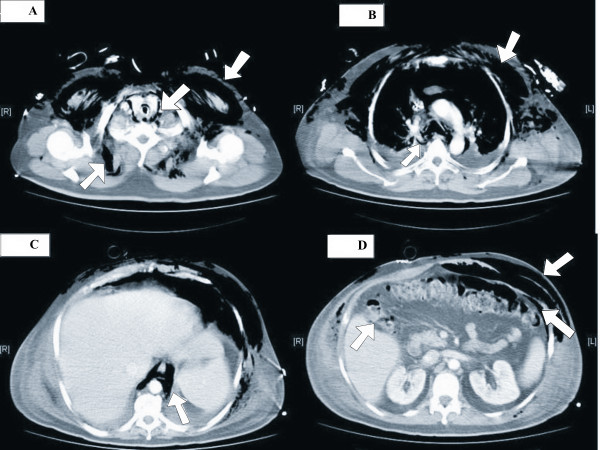
**Computed tomography images showing**: (A) Subcutaneous emphysema with retrosomatic (lower left arrow) and pectoral musculature gas dissection (upper right arrow), air in the infrahyoid visceral cervical space with separation of thyroid lobe vascular structures (central arrow); (B) Large subcutaneous emphysema in the anterior chest wall (upper right arrow). Gas dissection of the peribronchial cuff in the context of pneumomediastinum (lower left arrow); (C) Presence of air in the retrocrural space. Subcutaneous emphysema, mainly in the anterior chest wall. Pneumoperitoneum with perihepatic distribution (lower right arrow); (D) Subcutaneous emphysema with oblique muscle dissection and anterior pneumoperitoneum (upper right arrows). Small air bubbles below the vesicular bed (upper left arrow).

Computed tomography of the chest and abdomen revealed a large pneumomediastinum, pneumoretroperitoneum, gas in subcutaneous tissue and two flank abscesses (Figure 1). During surgical intervention, cutaneous and subcutaneous necrosis was observed, with muscular involvement. Abscess drainage was performed by two parallel incisions of the skin at 2 cm and 4 cm from the costal margin, followed by dissection of the subcutaneous tissue and the fascia of each flank, directly over each abscess at the point of greatest fluctuation. In both blood and surgical wound exudate cultures, *Escherichia coli *was found. This was treated with ciprofloxacin and piperacillin-tazobactan".

After prolonged hospitalization, the patient finally recovered and was discharged. At one year of follow up, the patient was well and showed no signs of relapse.

## Discussion

EC, also known as acute gaseous cholecystitis, aerocholecystitis, pneumocholecystitis, and gas gangrene of the gallbladder, is a severe variant of acute cholecystitis [2]. Welch and Flexner first reported EC pathologic findings in 1896. In 1901, Stolz described the presence of gas in the gallbladder after post-mortem autopsy of three patients. Hegner first described EC radiographic findings in 1931.

Anatomic pathology studies of EC gallbladders show a high incidence of endarteritis obliterans and vascular occlusion of the cystic artery. This is postulated to lead to an isquemic environment where gas-producing micro-organisms reproduce, resulting in gas penetrating the wall of the gallbladder [2]. This theory is supported by cases of EC reported after cardiopulmonary resuscitation with systemic hypoperfusion and after transhepatic arterial embolization resulting in embolization of the cystic artery [4].

Cultures obtained from EC gallbladders are positive for micro-organisms in 95% of cases, especially including *Clostridium spp, Escherichia coli*, and *Klebsiella spp *[1,2]. The fact that 50% of EC patients are diabetic may be due to features of the disease itself and associated comorbidity which favour ischemic environments and increased incidence of infection [2]. The increased frequency and severity of infection is directly related to metabolic state; thus in those patients with good control of glucemia levels, the incidence of infection is similar to that of the general population.

Greater susceptibility to infection in poorly controlled diabetic patients is attributable to the presence of hyperglucemia and acidosis, which result in reduced mobility of fagocytes in the areas of infection and reduced antimicrobial activity. Fagocyte destruction of germs is carried out by oxidative processes. Under normal conditions, these cells obtain sufficient quantities of NADPH to reduce oxygen supply by means of glycolisis. In situations where insulin is not available, the process of glycolisis is altered, leading to reduced antimicrobial capacity [5].

Alterations of the diabetic immune system have been postulated, including variations in subpopulations of lymphocyte T and reduced total populations of these cells, as well as lower concentrations of immunoglobulin [6].

Other factors favoring infection in some diabetic patients are the chronic complications of the disease itself. Diabetic polyneuropathy conditions a lower sensitivity to insult such as slight trauma and burns in the lower limbs, which may provide access and environment for infectious processes. Difficulty with urinary evacuation of the bladder due to autonomic nervous system neuropathy favors infection of the urinary pathways, while fecal incontinence, also related to this neuropathy, may provoke skin laceration and ulcers. In addition, diabetic arteriopathy gives rise to distal tissue ischemia, which increases the tendency to bacterial proliferation.

Among the complications of EC, dissemination to pericholecystic tissue or to the common bile duct is unusual, while subcutaneous gas has only been reported in isolated cases. The literature contains one case in which the emphysematous gallbladder was not perforated, with gas disseminated to the central retroperitoneum along the choledochus [3].

Anatomically, the retroperitoneum, mediastinum, back and neck are connected by fascial planes which allow migration of air due to pressure gradient. Cases have also been reported of subcutaneous emphysema of the lower limbs after emphysematous infection of the abdomen and pelvis. This connection between anatomic spaces may explain how retroperitoneal air produced by gas-producing micro-organisms reaches mediastinal tissue, and subcutaneous tissue of the back and neck, giving rise to pneumomediastinal and subcutaneous emphysema respectively [3,7].

The presence of pneumomediastinum suggests the presence of an underlying life-threatening disease. The main cause of pneumomediastinum is rupture of intrathoracic structures such as the esophagus, trachea, bronchi or alveoli, although there are other possible causes such as cervical or closed oral trauma, severe or obstructive pulmonary disease, and intoxication [8]. To our knowledge, the literature contains no studies reporting the occurrence of pneumomediastinum after open cholecystectomy, only after laparoscopic cholecystectomy [9]. We are unable to postulate a causal link between open cholecystectomy and pneumomediastinum. In our case, the presence of pneumomediastinum was attributed to the development of EC related to the diabetic status of the patient.

## Conclusion

EC should be considered as a possible cause of pneumomediastinum, especially in diabetic patients. Diagnosis should not be delayed given the importance of early treatment of this potentially fatal condition.

## Competing interests

The authors have no competing interests (political, personal, ideological, academic, intellectual, commercial or any other) to declare in relation to this manuscript.

## Authors' contributions

LD wrote the first draft and is the guarantor. IG and ET provided the images and wrote the figure legends. DR collected the data and was involved in the discussion. All authors read and approved the final manuscript.

## Consent

Written informed consent was obtained from the patient for publication of this case report and any accompanying images. A copy of the written consent is available for review by the Editor-in-Chief of this journal.

## Pre-publication history

The pre-publication history for this paper can be accessed here:

http://www.biomedcentral.com/1471-230X/10/99/prepub
